# Spatiotemporal Evolution and Driving Forces of PM_2.5_ in Urban Agglomerations in China

**DOI:** 10.3390/ijerph20032316

**Published:** 2023-01-28

**Authors:** Huilin Yang, Rui Yao, Peng Sun, Chenhao Ge, Zice Ma, Yaojin Bian, Ruilin Liu

**Affiliations:** 1 School of Geography and Tourism, Anhui Normal University, Wuhu 241002, China; 2 School of Geography, Nanjing Normal University, Nanjing 210023, China

**Keywords:** PM_2.5_, influence factors of PM_2.5_ concentration, driving forces, urban agglomeration

## Abstract

With the rapid development of China’s economy, the process of industrialization and urbanization is accelerating, and environmental pollution is becoming more and more serious. The urban agglomerations (UAs) are the fastest growing economy and are also areas with serious air pollution. Based on the monthly mean PM_2.5_ concentration data of 20 UAs in China from 2015 to 2019, the spatiotemporal distribution characteristics of PM_2.5_ were analyzed in UAs. The effects of natural and social factors on PM_2.5_ concentrations in 20 UAs were quantified using the geographic detector. The results showed that (1) most UAs in China showed the most severe pollution in winter and the least in summer. Seasonal differences were most significant in the Central Henan and Central Shanxi UAs. However, the PM_2.5_ was highest in March in the central Yunnan UA, and the Harbin-Changchun and mid-southern Liaoning UAs had the highest PM_2.5_ in October. (2) The highest PM_2.5_ concentrations were located in northern China, with an overall decreasing trend of pollution. Among them, the Beijing-Tianjin-Hebei, central Shanxi, central Henan, and Shandong Peninsula UAs had the highest concentrations of PM_2.5_. Although most of the UAs had severe pollution in winter, the central Yunnan, Beibu Gulf, and the West Coast of the Strait UAs had lower PM_2.5_ concentrations in winter. These areas are mountainous, have high temperatures, and are subject to land and sea breezes, which makes the pollutants more conducive to diffusion. (3) In most UAs, socioeconomic factors such as social electricity consumption, car ownership, and the use of foreign investment are the main factors affecting PM_2.5_ concentration. However, PM_2.5_ in Beijing-Tianjin-Hebei and the middle and lower reaches of the Yangtze River are chiefly influenced by natural factors such as temperature and precipitation.

## 1. Introduction

PM_2.5_ is a particle smaller than 2.5 microns, which is an important cause of smog. In addition, people’s health is significantly affected by fine particulate matter (PM_2.5_) [1]. The World Health Organization estimates that airborne pollution causes 4.2 million deaths globally each year [2]. Previous studies have found that long-term exposure to high PM_2.5_ concentrations is associated with respiratory, cardiovascular, and neurological disorders [3,4,5]. In the past forty years, China has experienced the rapid development of industrialization and urbanization and achieved a high degree of urban integration [6]. China has developed into several urban agglomerations (UAs) that are now significant engines of economic growth. Nevertheless, the high-speed development of an urban economy is accompanied by serious air pollution [7,8]. Air pollution emissions at the scale of UAs are more serious than those at the scale of cities [9]. Hence, it is essential to understand the spatial distribution of PM_2.5_ concentration in UAs and analyze the main factors affecting its distribution. This will provide a scientific basis for improving urban air quality, reducing people’s health risks, and promoting sustainable urban development.

Many researchers mainly analyzed the spatiotemporal variation of PM_2.5_ from the perspectives of economic zones, cities, and rural areas [10,11,12,13]. In addition, previous studies have also investigated the inhibitory and enhancing effects of small-scale areas, such as green areas, wetlands, and roads, on PM_2.5_ emissions [14,15,16]. The spatiotemporal distribution of PM_2.5_ has significant heterogeneity [17]. Due to poor dispersion circumstances, northern China experienced more PM_2.5_ haze occasions than southern China. Furthermore, eastern China experienced more air contamination than central and western China as a result of greater rates of urbanization [18,19]. Unbalanced economic development in Hubei Province lead to higher PM_2.5_ concentrations in the east, south and north than in the northwest and southeast [20]. In addition, there were significant differences in the distribution of PM_2.5_ in Beijing-Tianjin-Hebei. Regions such as Shijiazhuang and Hengshui, far from the ocean, had higher concentrations of PM_2.5_, whereas coastal cities had lower concentrations [21]. This finding was due to favorable conditions for air diffusion in coastal cities and the fact that atmospheric pollutants are easily spread.

Many scholars have studied the factors affecting PM_2.5_ concentration mainly in two aspects: natural factors and socioeconomic factors. [22]. Numerous studies have shown that temperature, humidity, wind speed, and vegetation area are the main natural factors affecting PM_2.5_ concentrations [23,24,25,26]. In addition, anthropogenic factors, including population density, economic growth, industrial manufacturing, and resource depletion, have a significant impact on PM_2.5_ [27,28]. Spatial distribution differences in PM_2.5_ may be attributed to regional differences in its driving factors [29]. Moreover, many researchers also divided China into different regions to reveal the different effects of PM_2.5_’s decisive factors at the regional scale in China [30,31].

In recent years, many studies have focused on the impact of China’s urban agglomeration development on PM_2.5_. These studies found that natural factors (precipitation, wind speed, temperature, elevation) and socioeconomic factors (GDP, population density, secondary industry, foreign investment, energy, urbanization rate, and urban construction) have a great influence on PM_2.5_ concentration [32,33,34]. Among them, PM_2.5_ concentration is negatively correlated with precipitation, wind speed and temperature and positively correlated with elevation, GDP, population density, secondary industry, energy, urbanization rate, and urban construction. Foreign investment can not only bring in polluting industries to exacerbate PM2.5 pollution but can also introduce advanced technologies to reduce pollution.

In summary, the overall study of 20 UAs in China is still insufficient, which hinders an integrated understanding of the spatial-temporal variation of PM_2.5_ concentrations [35]. PM_2.5_ concentration data at large scales have been less studied. In addition, studies on the comprehensive use of natural conditions and socioeconomic factors to investigate the causes and diffusion mechanism of PM_2.5_ concentration are insufficient. Therefore, this study analyzed the spatial and temporal distribution of PM_2.5_ pollution in 20 UAs in China using monthly PM_2.5_ pollution data from 2015 to 2019. Then, the influence of natural and social factors on the spatial heterogeneity of PM_2.5_ in different UAs was quantitatively revealed by geographic detectors. Compared with previous studies, this paper makes contributions in the following three aspects. First, using data from national environmental monitoring stations in 20 UAs, this paper summarizes the spatiotemporal evolution of PM2.5 from the perspective of UAs. Second, using the geographic detector model, the main natural and socioeconomic factors that affect the spatiotemporal differentiation of PM2.5 at large spatial scales are explored. Finally, we select the UAs with the most serious pollution, and analyze the change rule of the most important influencing factors of the UAs. The temporal and spatial trends can be fully explained by considering natural and socioeconomic factors. Additionally, the selected sample cities are sufficient, basically covering the typical areas of China. This paper will provide some scientific guidance for air pollution control and the construction of livable cities where people and nature live in harmony.

## 2. Study Area and Data

### 2.1. Study Area

**Figure 1:**
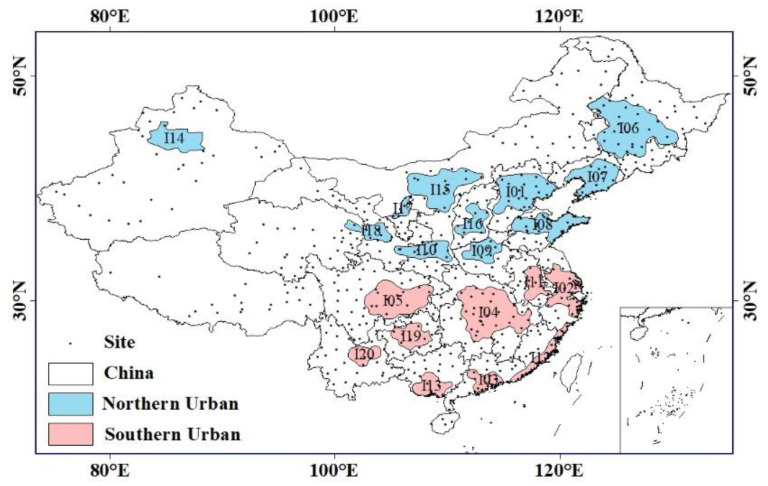
Map of the geographical location of UAs. (I01: BTH; I02: YRD; I03: PRD; I04: MRYR; I05: CC; I06: HC; I07: ML; I08: SP; I09: CH; I10: GZ; I11: JH; I12: WCTS; I13: BG; I14: NTM; I15: HBEY; I16: CS; I17: NYR; I18: LX; I19: CG; I20: CY).

This study focused on 20 UAs in China (Figure 1), namely the Beijing-Tianjin-Hebei (BTH), mid-southern Liaoning (ML), the Yangtze River Delta (YRD), Guanzhong (GZ), Pearl River Delta (PRD), central Henan (CH), the middle reaches of the Yangtze River (MRYR), central Guizhou (CG), Chengdu-Chongqing (CC), Harbin-Changchun (HC), the Shandong Peninsula (SP), Jiang-Huai (JH), the west coast of the Taiwan Strait (WCTS), the Beibu Gulf (BG), North Tianshan Mountain (NTM), Hu-Bao-E-Yu (HBEY), central Shanxi (CS), Ningxia Yellow River (NYR), Lanzhou-Xining (LX), and central Yunnan (CY). Among them, the five largest UAs in the country were the BTH, YRD, PRD, MRYR and CC UAs, and the four regional medium UAs were the HC, ML, GZ and NTM UAs.

### 2.2. Data

The study data included PM_2.5_ data (annual, monthly, and daily data for each city in China). According to previous studies [36], the source factors of air pollution need to consider both natural and socioeconomic factors. Among the natural factors, the most important meteorological factors affecting PM_2.5_ distribution are temperature (TMP) and precipitation (PR), and the most important topographic indicators are elevation (DEM) and slope (SL) [37]. Among the socioeconomic factors, total population at year-end (PD), GDP (GDP), secondary industry (IS), social electricity consumption (EC), civil vehicle ownership (CV), and foreign direct investment (FDI) were considered as socioeconomic factor data (Table 1). IS reflects the level of industrialization, and EC mainly reflects the degree of energy consumption. (1) PD: the increase in urban population will give rise to an increase in air pollutant emissions in production and living patterns [38]. (2) GDP: it reflects the stage of economic development, and different stages of economic development often show different energy consumption intensities [39]. (3) IS: industrial energy consumption by industrial activities is the main cause of the rise in PM_2.5_ concentrations [40]. (4) EC: electricity consumption is the main source of PM_2.5_ industrial emissions [41]. (5) CV: motor vehicle exhaust has become one of the crucial sources of urban atmospheric pollution [42]. (6) FDI: it changes the industrial structure and hinders the upgrading of industrial structure, wastes resources, and pollutes the environment [43]. Monthly PM_2.5_ data and daily data were obtained from the urban air quality release platform and the ecological and environmental quality bulletin of each province (https://www.aqistudy.cn/historydata/ (accessed on 12 December 2022)). Topographic data were obtained from the China Resources Science Records Center (https://www.resdc.cn/ (accessed on 12 December 2022)) [44]. Meteorological data were obtained from the China Meteorological Data Network (https://www.geodata.cn/ (accessed on 12 December 2022)). The annual mean dataset of each factor was obtained by raster calculation extraction and collation. PD, GDP, IS, EC, CV, and FDI data were from the local provincial and municipal statistical yearbooks of each region (http://www.tjcn.org (accessed on 12 December 2022)).

## 3. Methodology

### 3.1. PM_2.5_ Data Classification

According to GB3095-2012 Chinese Ambient Air Quality Standards (CAAQS), PM_2.5_ concentration can be classified into three levels, and the annual mean PM_2.5_ concentration is less than 15 ug·m^−3^ for the first level, between 15 ug·m^−3^ and 35 ug·m^−3^ for the second level and above 35 ug·m^−3^ for the third level.

### 3.2. Geographic Detectors

Geographic detectors are used to identify spatial heterogeneity and quantify its impact factors. It includes four detectors: risk area, interaction, variance and factor, and ecological detection. In this study, we centered on identifying the spatial dividing of PM_2.5_ by diverse factors and evaluating whether two factors together increment or diminish the information on PM_2.5_. Hence, we use variance factor and interaction detection to identify drivers of PM_2.5_ [45].

(1) Variance and factor detection: identified the spatial disparity of property Y and the illustrative power of factor X on property Y. *q* is calculated as follows:

(1) $$q=1-\frac{\sum_{h}^{L}N_{h}\sigma_{h}^{2}}{N\delta^{h}}=1-\frac{SSW}{SST}$$

(2) $$\mathrm{SSW}=\sum_{h=1}^{L}N_{h}\sigma_{h}^{2},\;SST\cdot N\cdot \sigma^{2}$$

where *q* is the explanatory degree of the PM_2.5_ impact factor, h is the classification of the PM_2.5_ concentration impact factor, *N_h_* and *N* are the impact factor h and the total number of impact factors, respectively, and $\sigma^{2}$ and $\sigma_{h}^{2}$ are the variance of the total sample size and the variance classification, respectively. Within the sum of squares (SSW) and the total sum of squares (SST) are the sum of variances within the classification and the total variance of the whole classification, respectively. The value range of Q is [0, 1], and the larger the number, the more obvious the spatial heterogeneity of Y. In the case where the independent variable X produces a classification, the larger the value of *q*, the larger the explanation of the variable X on the dependent variable Y, and vice versa. If the value of *q* is 1, it indicates that the spatial distribution of Y is mainly affected by the independent variable X. When the value of *q* is 0, it means that the independent variable X has no effect on Y. In addition, the *q* value indicates that the quantitative impact degree of the independent variable X on the dependent variable Y is 100 × *q*%.

(2) The interaction detection was the detection of the degree of two or more impact factors acting together on attribute Y. This method includes the following five relationships, which are detailed in Table 2.

## 4. Results

### 4.1. Temporal Variation Characteristics of PM_2.5_ Concentration

**Figure 2:**
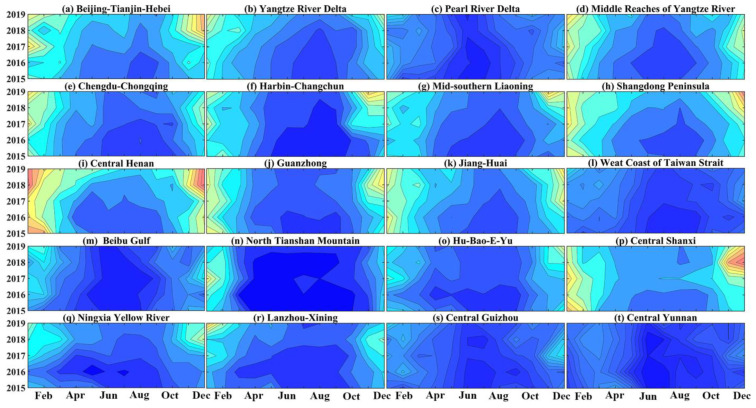
Annual and monthly variation characteristics of PM_2.5_ concentration in UAs from 2015 to 2019.

**Figure 3:**
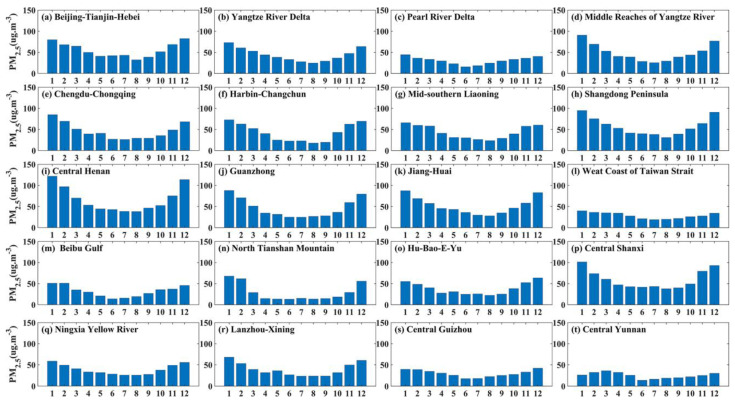
Mean PM_2.5_ mass concentration in each month from 2015 to 2019.

Figure 2 and Figure 3 show annual and monthly variation features of PM_2.5_ concentration in UAs. The monthly mean PM_2.5_ concentrations showed high values in November-January and the lowest values in June or July. The change curve of monthly mean PM_2.5_ concentration presents a “U”-shaped feature.

It is obvious from Figure 2 and Figure 3 that, among the five national UAs, the monthly PM_2.5_ concentrations of BTH were higher than those of the other 4 UAs. In 2018, the concentration of PM_2.5_ was the highest, up to 84.83 ug m^−3^. The monthly mean PM_2.5_ concentrations of YRD, MRYR, and CC were basically the same. PRD had the lowest PM_2.5_ concentration, with the maximum PM_2.5_ concentration around 45 ug·m^−3^. Among the four regional medium-sized UAs, high values of PM_2.5_ in HC and ML occur earlier in October (60.5 ug·m^−3^) and November (64.7 ug·m^−3)^, respectively. Among the other UAs, CH and CS were the most polluted in winter, and CY reached its highest value in March, with concentrations as high as 102.43 ug·m^−3^, 80 ug·m^−3^ and 38.43 ug·m^−3^.

### 4.2. Spatial Variation Characteristics of PM_2.5_ Concentration

**Figure 4:**
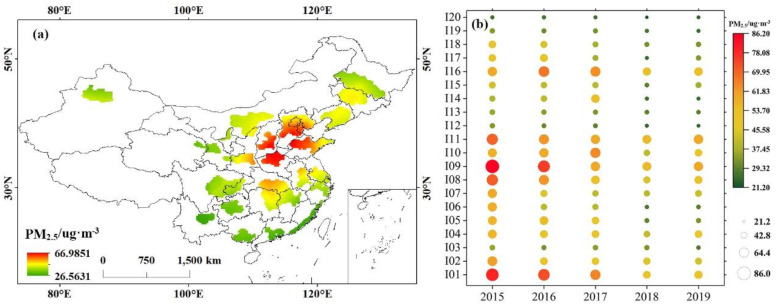
(**a**) Spatial distribution of mean annual PM_2.5_ concentration and (**b**) changes in PM_2.5_ concentration in different UAs in different years.

The spatial distribution of PM_2.5_ in UAs in China was uneven. The high mean PM_2.5_ concentration places in the country were chiefly located in the northern region such as BTH, SP, CS, and CH, with the highest values of 57.8 ug·m^−3^, 57.36 ug·m^−3^, 55.76 ug·m^−3^ and 56.21 ug·m^−3^. The annual mean PM_2.5_ concentration of these UAs exceeded the CAAQS secondary annual mean concentration standard in 2015–2019 (Figure 4a). However, the PM_2.5_ concentration in UAs showed a descent trend, especially in BTH (I01), SP (I08), and CH (I09), which indicates that the sequence of laws and regulations to deal with smog has achieved significant results in China, and air pollution control projects have made certain achievements in the past few years. 

**Figure 5:**
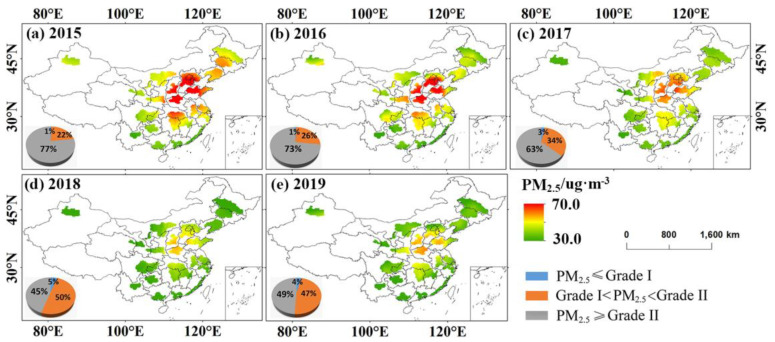
Spatial distribution of PM_2.5_ concentrations in different levels of PM_2.5_.

Figure 5 shows the distribution of annual mean PM_2.5_ concentration in 20 UAs from 2015 to 2019. From a geographical area perspective, the high-value area of annual PM_2.5_ concentration was consistent with Figure 4. The comprehensive comparison showed that the declining trend of PM_2.5_ concentration in the northern UAs (mainly BTH) was significantly higher than that in the southern UAs (mainly CY).

In 2015, mean annual PM_2.5_ concentrations in about 77% of air quality monitoring stations exceeded the CAAQS Level 2 limit, and only 1% of stations met the CAAQS Level 1 standard. From 2015 to 2019, the number of stations whose annual mean PM_2.5_ concentration met the first-level limit did not increase significantly. The station with an annual mean PM_2.5_ concentration exceeding the CAAQS secondary limit showed a decreasing trend within 5 years. However, by 2019, PM_2.5_ concentrations in about 47% of the station did not meet the CAAQS secondary level, and China’s PM_2.5_ control is still facing great challenges.

**Figure 6:**
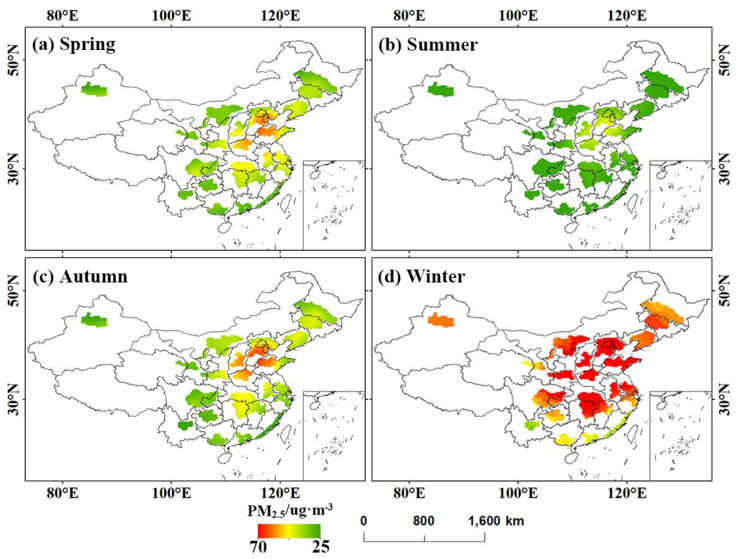
Spatial distribution of seasonal variation of PM_2.5_ concentration.

The spatial of seasonal mean PM_2.5_ concentration is demonstrated in Figure 6. PM_2.5_ was the most polluted in winter, with spring and autumn following, and summer air quality was optimal. In addition, compared to CY, BG and WCTS, other major UAs had serious PM_2.5_ pollution in winter. In the degree and extent of pollution, spring and autumn were significantly reduced compared with winter, though BTH, SP, CS, and CH were relatively polluted areas. PM_2.5_ concentrations in some areas of BTH, SP, CS, CH, ML, and HC were higher in autumn than in spring, while in other areas, spring was higher than autumn. The PM_2.5_ concentration in UAs was low in summer, and the air quality was best throughout the year. However, the PM_2.5_ high-value area was still distributed in BTH, SP, CS, and CH.

### 4.3. Analysis of Impact Factors on PM_2.5_ Concentration

#### 4.3.1. Factor Detection

In Table 3, all factors in each year passed the hypothesis test at the 5% level. The explanatory powers of X8 (EC), X9 (CV), and X10 (FDI) factors on PM_2.5_ concentration in UAs were stronger, and the explanatory degree of socioeconomic factors on PM_2.5_ concentration was stronger than natural factors.

**Figure 7:**
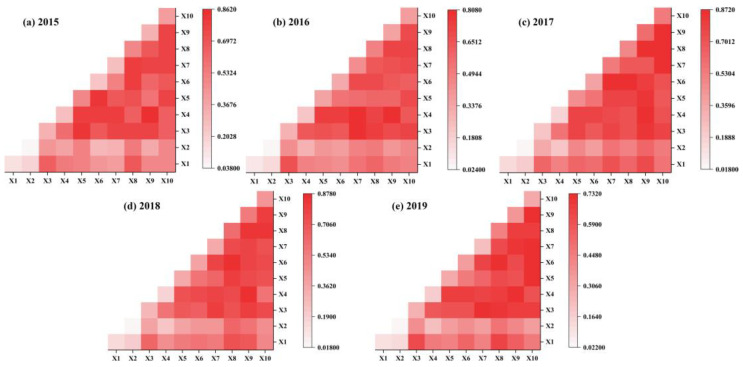
Change factor interaction detection results. (X1: DEM; X2: SL; X3: TMP; X4: PR; X5: GDP; X6: PD; X7: IS; X8: EC; X9: CV; X10: FDI).

Figure 7 shows that the interaction results of each year are two-factor enhancements or nonlinear enhancements; there is no nonlinear weakening and independent situation. Changes in the PM_2.5_ concentration are the outcome of the interaction of different factors, and interactions between various types of factors have different degrees of enhancement of the single factor of PM_2.5_ concentration change.

In 2015 and 2016, X4 (PR) had the greatest impact on PM_2.5_ when combined with other socioeconomic factors. Hence, natural factors acting as “catalysts” could boost the explanations for the effects of other social variables on PM_2.5_ concentrations. From 2017 to 2019, socioeconomic factors contributed to the comprehensive control of air pollution.

#### 4.3.2. Analysis of Impact Factors on PM_2.5_ in Typical UAs

**Figure 8:**
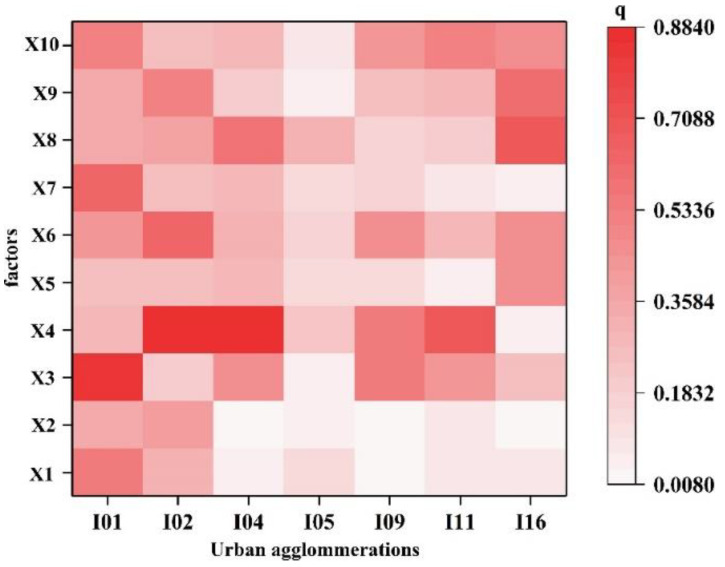
The impact degree in typical urban agglomeration factors. (X1: DEM; X2: SL; X3: TMP; X4: PR; X5: GDP; X6: PD; X7: IS; X8: EC; X9: CV; X10: FDI; I01: BTH; I02: YRD; I04: MRYR; I05: CC; I09: CH; I11: JH; I16: CS).

Based on the above research results, seven UAs, including CH (I09), BTH (I01), JH (I11), CS (I16), YRD (I02), MRYR (I04) and CC (I05), are heavily polluted. Hence, we selected seven UAs to study the impact factors. According to Figure 8, the main factors affecting BTH were X3 (TMP), X7 (IS), and X10 (FDI), while X4 (PR) had a significant effect on MRYR, YRD, and JH. YRD was mainly influenced by social factors X6 (PD) and X9 (CV). CS and CC were mainly affected by X8 (EC), and CH was mainly influenced by X6 (PD).

## 5. Discussion

The temporal distribution characteristics of 20 UAs in China showed the most serious pollution in winter. The winter season has less rainfall than other seasons, and the air lacks the flushing of rainwater; thus, fine particles readily float in the air [46,47]. Moreover, the lower mean temperature in winter tends to create a steady atmospheric stratification, which is not conducive to contaminant dispersion [48]. The steady atmospheric perpendicular structure undermines the turbulent atmospheric exchange and convection of heat, which impedes the attenuation and diffusion of contaminants in the perpendicular direction, leading to the higher viscosity of contaminants in the air [49]. In addition, the large increase in coal combustion in winter has led to a great increase in PM_2.5_ emissions in the areas north of the Yangtze River [50,51]. In contrast, the southwestern CY and southern coastal areas of BG and WCTS UAs had lower PM_2.5_ concentrations in winter. In terms of topographic factors, more mountainous areas are prone to local convection. Meteorological factors such as high temperatures make the vertical movement of the atmosphere more active, and, thus, turbulent action occurs near the ground. In addition, the effect of the sea and land breezes in coastal areas leads to better diffusion conditions [52].

Among the five national UAs, the PM_2.5_ concentration each month in BTH was much higher than the PM_2.5_ concentration in other UAs. Among the influencing factors, secondary industry, foreign investment, and temperature were the most important influencing factors. Secondary industry power consumption accounts for 52.1% of the comprehensive power consumption; energy consumption is much higher than other industries, and industrial production chiefly depends on coal. Therefore, massive pollutants produced by coal consumption straightforwardly exacerbate air pollution [40]. On the other hand, in the Beijing-Tianjin-Hebei region, foreign investment tends to be in the tertiary industry, transforming the industrial structure, especially in developing economies, which may affect the transfer and allocation of resources between different industries. The irrational industrial structure will distort the allocation of factors, increase the waste of resources, and produce a lot of pollution. Energy consumption due to the rapid development of tertiary industry in big cities is also expanding. The proportion of tertiary industry energy consumption in the Beijing-Tianjin-Hebei region increased by 22.4% in the past five years. The upgrading of the industrial structure will reduce the PM_2.5_ concentration, but the introduction of foreign investment, with manufacturing taking the highest proportion, has hindered the upgrading of the urban industrial structure [53]. This has exacerbated the PM_2.5_ contamination status in the Beijing-Tianjin-Hebei region. The temperature in BTH is on a steady decline in winter, and the convective movement of the atmosphere is weakened. The pollutants are not easy to diffuse, giving rise to higher PM_2.5_ concentrations [54]. BTH UA showed exceptionally high values of PM_2.5_ in 2018, and economic growth may be the chief factor for the increase in PM_2.5_ concentration in 2018 [55]. 

However, the middle and lower reaches of the Yangtze River (YRD, MRYR, and JH UAs) were most affected by precipitation. The YRD, MRYR, and JH UAs have a subtropical monsoon climate with simultaneous rain and heat. Humidity is greater than that in other regions, which is conducive to the hygroscopic growth of PM_2.5_ concentrations. Meanwhile, the high humidity state is more beneficial to the effective removal of airborne particulates [47]. Population and motor vehicle ownership were the most prominent social influences in the YRD UA. The 2015–2019 YRD population census showed a significant upward trend, with the highest value reaching more than 220 million people, and the population density of each central city was greater than 15,000 people/km^2^. The PM_2.5_ population exposure is also high in areas with high population density. Hence, the concentration of the YRD population distribution has a greater impact on PM_2.5_ concentrations [56]. YRD motor vehicle ownership reached more than 8 million, and motor vehicle fuel consumption was 16.6 million tons. During the “blowout” period of the rapid growth of the YRD from 2015 to 2019, fuel consumption increased rapidly, aggravating PM_2.5_ contamination in the Yangtze River Delta region [57]. The CC UA was mainly influenced by social electricity consumption. In recent years, the proportion of traditional heavy industries such as steel, cement, glass, and ceramics in industrial value added has increased, and the increase in heavy industries will consume more electricity, which in turn aggravates the pollution in the CC region [58].

Among the four territorial secondary UAs, the HC and ML UAs started to show high values in October and November. Because these places are situated in the northeast, the annual heating stage is early, and massive particulate matter from burning owing to centralized heating is emitted into the air. Furthermore, the northeast is an important commercial grain base in China, and October and November are the autumn harvest season in Northeast China. There is a large amount of open-air burning of straw, and Heilongjiang Province ranks first in the number of fire spots among all provinces [59]. Hence, the peak of burning occurs in November, and the particulate matter generated by crop residue burning and the secondary organic aerosols it forms cause local and regional degradations in atmospheric quality [60].

In other UAs, PM_2.5_ in the SP UA was the highest. From the perspective of meteorological conditions, cold air activity is weak in autumn and winter, and the northern region has high temperatures and little rain, which leads to poor conditions for the diffusion of air pollution. Disadvantageous meteorological conditions such as heat, light wind, low boundary layer, and frequent temperature inversions cause the rapid accumulation of pollutants. For internal factors, dust pollution control is still extensive in some areas; there are still weak links in the control of various types of dust, such as construction, roads, and industrial enterprise fugitive emissions, and the level of urban fining management is low [61]. The difference between PM_2.5_ concentration between the CH and CS UAs in winter and summer was obvious. The CH UA has a low surface temperature in winter, stable airflow, and poor vertical diffusion conditions in the atmosphere. It is easy to form an inversion effect, which is not favorable to the diffusion and degradation of PM_2.5_ [62]. However, rainfall occurs frequently in summer and has a certain erosion effect on the particulate matter in the atmosphere [63]. The CH UA is situated in the North China Plain and is densely populated, so its strongest anthropogenic factor is population [64]. The CS UA is affected by winter climate characteristics, winter heating and other pollutant emissions, corresponding to the weather situation for static pollution [65]. Among the human factors, social electricity consumption has the greatest impact on the CS UA. The CS UA is dominated by thermal power generation. PM_2.5_ produced by thermal power generation is the largest source of emissions in industry; part of this particle pollution comes from dust in flue gas, and the other part comes from PM_2.5_ aerosols produced by the reaction of nitrogen oxides and sulfides emitted in the air by coal combustion [66]. The CY UA’s concentrations reached their highest values in March, which is related to the burning of spring seeds in neighboring Southeast Asia, where pollutants from the combustion of substances enter the air and enter Yunnan Province through wind action, resulting in widespread air pollution [54]. The GZ UA showed the characteristics of “rising to fall”. The Ministry of Environmental Protection first included the GZ area as a significant area for air contamination prevention and control in early 2018, so the GZ UA air pollution was also controlled.

PM_2.5_ concentrations declined from 2015 to 2019, and especially, the declining trend of PM_2.5_ in the BTH UA is the most remarkable. Since 2015, the Chinese government has invested a lot of energy and resources in PM_2.5_ to focus on BTH UA, and the government is actively promoting coal substation plans [67]. The decline of the northern UAs (mainly BTH UA) was more obvious. The concentration base of pollution in the northern UAs is higher; thus, the decline in centralized control is higher than that in southern UAs [68].

In conclusion, this study objectively describes the spatial and temporal distribution of PM2.5 in 20 UAs during 2015–2019 and quantitatively reveals the impact of natural factors and socioeconomic factors on PM_2.5_ pollution using the geographic detector method. Although our research data comes from 20 urban agglomerations, our research methods and framework can be applied to pollution prevention and control efforts in other areas. Based on the above conclusions, China should further promote the coordinated treatment of PM2.5, carry out air pollution actions and improve coordination mechanisms. At the same time, local governments should formulate corresponding emission reduction policies according to their economic development level, industrial structure, energy consumption and other actual conditions so as to achieve collaborative governance with surrounding areas.

The factors influencing PM_2.5_ in UAs are very complex, and the air pollutants are complex and long-term. The influence of urban economic growth and urbanization expansion on air pollution is worth discussing. The public outcry over air pollution is also a future focus and needs to be explored in depth. Finally, it is also important to explore empirical studies of PM_2.5_ on public health, which can deepen our understanding of the disastrous consequences of deteriorating air quality in China.

## 6. Conclusions

This study objectively analyzed the spatiotemporal distribution characteristics of PM_2.5_ in 20 UAs in China from 2015–2019 and identified the main factors that affect the distribution of PM_2.5_ from the aspects of nature and socioeconomics. The main conclusions are as follows:

1. Due to low precipitation (and temperatures) in winter and a significant increase in coal burning, UAs were most polluted in winter, followed by spring and autumn, and the least polluted in summer. Among them, the central Henan and the central Shanxi UAs have the highest PM_2.5_ concentrations. However, the PM_2.5_ concentration in the central Yunnan UA was the highest in March, and the highest value in the Harbin-Changchun UA occurred in October.
2. Unlike most UAs where pollution was severe in winter, the central Yunnan, Beibu Gulf, and the West Coast of the Strait UAs had lower PM_2.5_ concentrations. These UAs have a large proportion of mountain areas and high temperatures, and they are easily influenced by land and sea breezes, which make pollutants more conducive to diffusion.
3. The spatial distribution of PM_2.5_ in UAs demonstrated an overall decreasing trend, especially in the Beijing-Tianjin-Hebei UA. PM_2.5_ high-value areas are mainly located in the Beijing-Tianjin-Hebei, central Shanxi, central Henan, and Shandong Peninsula UAs in northern China, with mean concentrations of 63.458 ug·m^−3^, 67.054 ug·m^−3^, 59.332 ug·m^−3^ and 56.62 ug·m^−3^, respectively, which is 42.8%, 50.9%, 33.5%, and 27.4% higher than the national mean PM_2.5_.
4. The effect of socioeconomic factors on PM_2.5_ concentration is stronger than that of natural factors. Moreover, three of the social factors, namely, social electricity consumption, civilian vehicle ownership, and use of foreign investment, have a great impact on the concentration of PM_2.5_ in UAs. There was obviously spatial heterogeneity in the drivers of PM_2.5_, and natural factors such as temperature and precipitation play a dominant role in Beijing-Tianjin-Hebei and the middle and lower reaches of the Yangtze River. Most UAs were affected by socioeconomic factors. Social electricity consumption was the main influencing factor in the central Shanxi UA and the Chengdu-Chongqing UA. The central Shanxi UA is dominated by thermal power generation, and the PM_2.5_ aerosol produced by coal combustion makes it seriously polluted. The values seen for the Chengdu-Chongqing UA chiefly resulted from the increase in heavy industries, which consume more electricity. The central Henan UA was mainly influenced by population.

## Figures and Tables

**Table 1:** Influencing factors and abbreviations.

Factors	Shorthand	Number
Elevation (m)	DEM	X1
Slope	SL	X2
Temperature (°C)	TMP	X3
Precipitation (mm)	PR	X4
GDP (billion)	GDP	X5
Total population at year-end (million)	PD	X6
Secondary industry (billion)	IS	X7
Social electricity consumption (kw·h)	EC	X8
Civil vehicles ownership (million)	CV	X9
Foreign direct investment (billion dollars)	FDI	X10

**Table 2:** Type of interaction between two independent variables and dependent variables.

Rules-Based	Effect
q(X_m_,∩X_n_) < min(q(X_m_),q(X_n_))	Nonlinear debilitating
min(q(X_m_),q(X_n_)) < q(X_m_,∩X_n_) < max(q(X_m_),q(X_n_))	Single-factor nonlinear weakening
q(X_m_,∩X_n_) > max(q(X_m_),q(X_n_))	Two-factor upgrade
q(X_m_,∩X_n_) = q(X_m_) + q(X_n_)	Independent
q(X_m_,∩X_n_) > q(X_m_) + q(X_n_)	Nonlinear enhancements

Note: q(X_m_,∩X_n_): the value of the interaction of two factors; min(q(X_m_),q(X_n_)): the minimum influence of two factors; max(q(X_m_),q(X_n_)): the maximum influence of two factors; q(X_m_), q(X_n_): single factor influence degree.

**Table 3:** Degree of factor influence.

Year	X1	X2	X3	X4	X5	X6	X7	X8	X9	X10
2015	0.133	0.038	0.313	0.275	0.501	0.241	0.271	0.499	0.328	0.415
2016	0.093	0.024	0.311	0.219	0.343	0.325	0.423	0.469	0.342	0.373
2017	0.149	0.018	0.239	0.165	0.458	0.367	0.542	0.488	0.611	0.531
2018	0.153	0.019	0.279	0.178	0.346	0.390	0.362	0.614	0.551	0.432
2019	0.096	0.023	0.275	0.176	0.286	0.347	0.232	0.428	0.374	0.301
mean	0.125	0.024	0.283	0.203	0.387	0.334	0.366	0.499	0.441	0.410
*p*	<0.001	<0.001	<0.001	<0.001	<0.001	<0.001	<0.001	<0.001	<0.001	<0.001

Note: X1: DEM; X2: SL; X3: TMP; X4: PR; X5: GDP; X6: PD; X7: IS; X8: EC; X9: CV; X10: FDI.

## Data Availability

The data presented in this study are available on request from the corresponding author.
